# Treatment of Incontinence of Urine in Women

**Published:** 1881-09

**Authors:** 


Treatment of Incontinence of Urine in Women.—Dr. J. Milner Chapman, M.B., M.R.C.S., Assistant to the Physician for Diseases of Women, Royal Infirmary, Edinburgh, read before the Edinburgh Obstetrical Society a paper, given in the Edinburgh *Medical Journal*, containing the following case: Mrs. C., age fifty-eight, complained of frequent and painful micturition, which had lasted three and a half years. When she first took ill a doctor told her she had inflammation of the bladder and some urethral affection (caruncle?), for both of which he treated her.

On September 30th, 1880, it was found that she could only retain water half an hour. The pudenda were reddened, as was also the whole vagina. The urethra was somewhat gaping at its outlet. There was considerable pain evinced on rubbing the two walls of the bladder over one another, or on introducing the sound into the viscus. The urine was turbid, acid, and contained puss cells, bladder epithelium, and some oxalates. The urethra was dilated by the finger, with the result of increasing the bladder's retaining limit to one and a half hours. At the same time nux vomica and uva ursi were given, and the vaginitis treated by sedative applications. The good effects of the dilitation disappeared in about three weeks, and it was then repeated, but soon she again relapsed into her former condition, minus, however, the pain and the presence of pus in the urine. The urethra was examined by the endoscope, and a slight redness was noticed. Iodoform bougies were used. The condition of the bladder wall, as seen by the endoscope, was normal, and now (November 8th) her only trouble was that every hour, day and night, she has to empty her bladder. The total quantity of urine passed was fifty ounces, which gave little more than two ounces as the amount at each micturition. The sound passed into the bladder three inches from the external meatus, and it was found that it could only be pushed half an inch further, and that thus pain was caused. It occurred to me that gradual forcible dilitation of the bladder might relieve the patient, and, with Dr. Augus Macdonald's permission, I proceeded to submit this idea to trial. The bladder was distended with warm two-per-cent. carbolic solution, and the quantity used measured and found to be four ounces. Any attempt to inject more caused the most intense pain, and the resistance offered was great, as could be felt by the difficulty experienced in compressing the ball of the syringe. From this date the bladder was filled to distention daily, the injection being stopped when the patient's pain became great, and when resistance reached a high point. The apparatus used was a Higginson's syringe attached to an ordinary catheter, care being taken to prevent the access of air to the bladder. Each day there was a gradual increase in the amount injected, of from a drachm to an ounce. On two or three occasions the fluid as it returned was tinged with blood, but no harm ensued. On December 20th she was discharged in the following condition: Instead of micturating every hour, she had only to get up once or twice during the night, and by day she could retain water so well as not to be inconvenienced. Sixteen ounces could now be injected into the bladder, and less pain was thereby caused than formerly, when four ounces was the limit. The urine was normal in all respects. Two months later she reported herself as being still as well as when she left hospital.

It will be seen from the case that the woman had a cystitis, with frequency of micturition, which latter remained after the former was cured; that any indication there was for further treatment was attended to either medicinally, topically, or by operation, but that still the frequent micturition continued; that the bladder was then found smaller than normal, both by measurement with the sound, and by the much more certain method of measuring its capacity, and that this capacity was increased fourfold by what may be called *slow operative dilitation of the bladder*, and that the results were in all respects satisfactory.

